# Precocious puberty: a comprehensive review of diagnosis and clinical presentation, etiology, and treatment

**DOI:** 10.2478/abm-2025-0009

**Published:** 2025-04-30

**Authors:** Khomsak Srilanchakon, Vichit Supornsilchai, Suttipong Wacharasindhu, Martin O. Savage

**Affiliations:** 1 Division of Pediatric Endocrinology, Department of Pediatrics, Faculty of Medicine, Chulalongkorn University, Bangkok 10330, Thailand; 2 School of Global Health, Faculty of Medicine, Chulalongkorn University, Bangkok 10330, Thailand; 3 Centre for Endocrinology, William Harvey Research Institute, Queen Mary, University of London, Charterhouse Square, London EC1M 6BQ, United Kingdom

**Keywords:** central precocious puberty, gonadotropin releasing hormone agonist, peripheral precocious puberty, precocious puberty

## Abstract

Central precocious puberty (CPP) is characterized by early activation of the hypothalamic–pituitary–gonadal (HPG) axis, which is apparent in the form of breast development in girls and testicular enlargement in boys prior to the typical physiological age ranges. Although intracranial pathology, exposure to high levels of sex steroids, or environmental risk factors can precipitate CPP, the majority of cases are idiopathic. Monogenic causes have also been identified. We provide a concise summary of the pathophysiology, risk factors, diagnosis, and management of CPP in this review. A referral to pediatric endocrinology should be initiated when there is concern for CPP. The diagnosis is confirmed through clinical, biochemical, radiological, and genetic testing. The primary objectives of administering a gonadotropin-releasing hormone (GnRH) analog to patients with CPP are to increase adult height and postpone the development of secondary sexual characteristics until a later age that is more compatible with peer norms. Although the long-term results of treatment with GnRH analogs are encouraging, further research is required to investigate the psychological impact of CPP.

The prevalence of precocious puberty is on the rise globally. Earlier studies in Asia disclosed that the documented surge in central precocious puberty (CPP) incidence in Korea, which commenced in the early 2000s, has persisted up to 2020. The annual prevalence of CPP has seen a significant escalation, with rates increasing from 2.7 to 206.5 (76.5 times) per 100,000 persons in boys and from 141.8 to 3439.9 (24.3 times) per 100,000 persons in girls, respectively [1]. This condition can greatly impact the physical and psychological well-being of both the patients and their families.

There are several long-term effects that are particularly concerning when it comes to early onset of menstruation in young girls. These effects include potential height loss and an increased risk of future complications such as obesity, diabetes, hypertension, hyperlipidemia, cardiovascular diseases, breast cancer, and stress [2]. General practitioners must possess a comprehensive knowledge base in order to provide accurate and appropriate primary care services. CPP is a frequently seen form, while peripheral precocious puberty (PPP) is comparatively rare. Both require specialized expertise for effective management and treatment.

## Normal physiology of pubertal control

**Figure 1.**
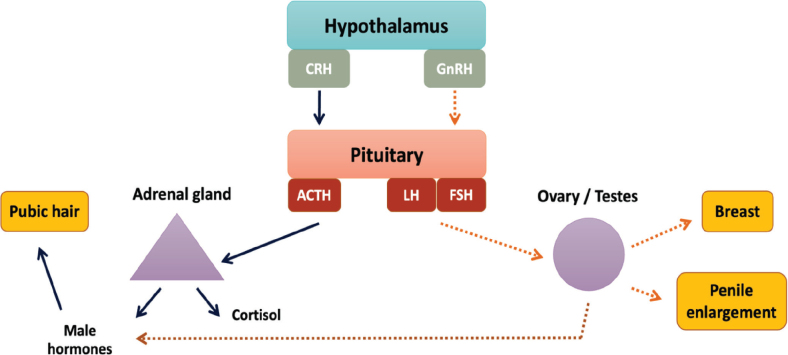
Development of the HPA and HPG axes during adolescence. ACTH, adrenocorticotropic hormone; CRH, corticotrophin-releasing hormone; FSH, follicular stimulating hormone; GnRH, gonadotropin-releasing hormone; HPA, hypothalamic–pituitary–adrenal; HPG, hypothalamic–pituitary–gonadal; LH, luteinizing hormone.

The onset of puberty is regulated by two important systems in the body: the hypothalamic–pituitary–gonadal axis (HPG axis) and the hypothalamic–pituitary–adrenal axis (HPA axis). Typically, the HPA axis is activated before the HPG axis [3] (Figure 1).

1. HPA axis: this axis stimulates the adrenal glands to produce adrenal androgens, originating in the zona reticularis under the hypothalamic influence. The process begins with the hypothalamus secreting corticotrophin-releasing hormone (CRH), stimulating the pituitary gland’s corticotrope cells to release adrenocorticotropic hormone (ACTH). ACTH further stimulates the adrenal glands, leading to the development of pubic hair and body odor in both boys and girls.
2. HPG axis: this axis triggers the ovaries in girls to produce estrogen and the testes in boys to produce testosterone. Initiated in the hypothalamus, the onset of puberty is controlled by stimulating factors such as kisspeptin (KISS1), glutamate, and neurokinin B, alongside inhibitory factors such as makorin ring finger 3 (MKRN3), gammaaminobutyric acid (GABA), and delta-like homolog 1 (DLK1) [4, 5]. When hypothalamic stimulation surpasses inhibition, gonadotropin-releasing hormone (GnRH) is secreted, activating the gonadotrophs in the pituitary gland. This leads to release of the luteinizing hormone (LH) and follicular stimulating hormone (FSH), stimulating the theca cells and granulosa cells of the ovary to secrete estrogen, and the Leydig cells in the testes to secrete testosterone.

During adolescence, the development of boys and girls diverges significantly. Sexual development can be classified using the Tanner stage classification system. In girls, this includes breast development (Table 1) and pubic hair growth (Table 2). For boys, it involves genital development (Table 3) and pubic hair growth (Table 4). Boys’ development also involves the measurement of testicular size using a tool called the Prader orchidometer. When testicular size is >4 mL or if the length exceeds 2.5 cm, the boy is considered to have entered puberty.

**Table 1.** Tanner classification of breast development in girls [6]

Tanner	Characteristics
1	Prepubertal: elevation of papilla only
2	Breast bud stage: elevation of breast and papilla as a small mound. Enlargement of areola diameter
3	Further enlargement and elevation of breast and areola, with no separation of their contours
4	Projection of areola and papilla to form a secondary mound above the level of the breast
5	Mature stage: projection of papilla only, due to recession of the general contour of the breast

**Table 2.** Tanner classification of pubic hair in girls

Tanner	Characteristics
1	Prepubertal
2	Sparse growth of long, slightly pigmented downy hair, straight or slightly curled, chiefly along labia
3	Considerably darker, coarser, and more curled. The hair spreads sparsely over the junction of the pubes
4	Hair now adult in type, but area covered is still considerably smaller than in the adult. No spread to the medial surface of the thighs
5	Adult in quality and type with distribution of the horizontal (or classically “feminine”) pattern. Spread to medial surface of thighs but not up linea alba or elsewhere above the base of the inverse triangle (spread up linea alba occurs late and is rated as stage VI)

**Table 3.** Tanner classification of genital development in boys [7]

Tanner	Characteristics
1	Prepubertal
2	Enlargement of scrotum and testes. Skin of scrotum reddens and changes in texture
3	Enlargement of penis, which occurs at first mainly in length. Further growth of testes and scrotum
4	Increased size of penis with growth in breadth and development of glans. Testes and scrotum larger; scrotal skin darkened
5	Genitalia adult in size and shape

**Table 4.** Tanner classification of pubic hair in boys

Tanner	Characteristics
1	Prepubertal
2	Sparse growth of long, slightly pigmented downy hair, straight or slightly curled, chiefly at the base of the penis
3	Considerably darker, coarser, and more curled. The hair spreads sparsely over the junction of the pubes
4	Hair now adult in type, but area covered is still considerably smaller than in the adult. No spread to the medial surface of the thighs
5	Adult in quality and type with distribution of the horizontal (or classically “feminine”) pattern. Spread to medial surface of thighs but not up the linea alba or elsewhere above the base of the inverse triangle (spread up linea alba occurs late and is rated as stage VI)

## Normal sequence of pubertal growth in boys and girls

In girls, puberty begins with breast development (Tanner stage 2). Subsequently, there is a rapid growth spurt, with a height increase of approximately 8–10 cm/year, starting approximately 2–3 years after the onset of breast development. Menstruation usually begins around age 11.5–13 [8].

In boys, puberty starts with testicular enlargement to approximately 4 mL. Then, there is a rapid growth spurt, with a height increase of approximately 10–12 cm/year, typically beginning 1–2 years later, when testicular enlargement reaches 8–10 mL. Acne and voice changes also occur.

It is noteworthy that girls enter puberty earlier than boys, but boys experience a greater amplitude growth spurt, leading to their eventual taller stature of approximately 13 cm.

The definitions of precocious puberty are as follows [9]:

For girls:

1. Development of breast (breast Tanner 2) before the age of 8 years.
2. Menstruation before the age of 9.5 years.

For boys:

1. Development of testes size over 4 mL or a length >2.5 cm before the age of 9 years.
2. Increase of phallus length or width before the age of 9 years.

The current height upon entering puberty early is often taller than the height calculated from the family’s average height (mid-parental height [MPH]). This can result from advanced bone age but may lead to a loss of adult height [10].

Mid-parental height in boys =

[(Father’s height + Mother’s height + 13) / 2] ± 8.5 (2 SDS)

Mid-parental height in girls =

[(Father’s height + Mother’s height - 13) / 2] ± 8.5 (2 SDS)

The diagnosis of precocious puberty involves several important steps:

1. Medical history [11]
  - *Past and recent growth patterns*: helps determine the starting point of early puberty.
  - *Pregnancy-related issues*: premature birth and certain brain conditions might trigger early puberty.
  - *Dietary habits*: nutritional factors could impact puberty timing.
  - *Developmental history*: some medical conditions might cause delayed development, such as hypothyroidism.
  - *Family history*: parents’ heights are used to calculate MPH, a reference for expected final height.
  - *Puberty onset in parents*: genetic factors could influence early puberty.
2. Physical examinations [12]
  2.1 Height measurement and national growth chart
  2.2 Physical examinations (Table 5)
3. *Bone age X-ray*: performed on the left hand and wrist, is a crucial tool for diagnosing of precocious puberty. Two methods, Greulich and Pyle, and Tanner Whitehouse, are used for evaluation. If the bone age is >2 standard deviations or 2 years above the actual age, it indicates advanced bone maturity, often seen in precocious puberty cases. If bone age is delayed compared with actual age, coupled with early puberty signs, other conditions such as thyroid hormone deficiency should be considered. Bone age X-ray is also vital for predicting adult height [13].
4. *Ultrasound*: in girls, ultrasound assists in examining the uterus and ovaries to identify precocious puberty causes. Enlarged uterus, especially with a pear-shaped appearance, may suggest precocious puberty. Examination of ovarian features can differentiate between central and PPP. If the ovaries are >1.5 cm and contain small cysts (follicular cyst), CPP is likely. In boys, ultrasound is not universally recommended but can be considered when irregular testicular masses or unequal testicular sizes are observed, particularly to examine Leydig cell tumors which may not be visible on ultrasound [14].
5. *Laboratory tests and hormone assessments*
  5.1 Basal gonadotropin and sex hormone tests: basal gonadotropin assays are essential for diagnosing precocious puberty. Current assays are highly sensitive and accurate. Elevated LH levels (≥0.3 IU/L) in girls are often associated with peak LH levels (≥5–7 IU/L) in GnRH stimulation tests. However, basal FSH levels are not useful for diagnosing precocious puberty. In boys, there is no definitive cut-off for basal LH to diagnose CPP. Our study demonstrates that the combination of basal LH (with a cutoff ≥0.2 IU/L) and the basal LH/FSH ratio (with a cutoff ≥0.1) can provide a straightforward and cost-effective method for diagnosing CPP in girls at breast Tanner stage II [15]. Basal sex hormone levels, especially estradiol in girls, are not routinely used due to frequent fluctuations. However, significantly elevated estradiol levels (>100 pg/mL) may indicate ovarian cysts or tumors. Morning testosterone levels in boys are useful, given the diurnal variation of testosterone, with the highest levels in the morning.
  5.2 GnRH stimulation test: the GnRH stimulation test involves administering a GnRH agonist, such as triptorelin (100 micrograms), subcutaneously. LH, FSH, and estradiol are measured at baseline and then every 30 min for 120 min. Peak LH levels exceeding 5–7 IU/L suggest CPP. If LH and FSH are suppressed post-test, it indicates PPP. If LH increases but remains below 5–7 IU/L, and FSH increases significantly, premature thelarche is diagnosed [16].
  5.3 Beta-human chorionic gonadotropin (HCG) and alpha-fetoprotein blood (AFP) tests: these tests are performed to detect conditions leading to PPP, checking if the patient’s condition involves the production of beta-HCG and AFP.
6. *MRI examination of the brain*: MRI of the brain is recommended for all cases diagnosed with CPP in boys, girls with neurological abnormalities, and girls exhibiting early puberty before the age of 6. Studies have shown that MRI can detect abnormalities, such as hypothalamic hamartoma, in the brain in cases of CPP in boys and young girls [17].
7. *Differential diagnosis*: the initial differential diagnosis involves medical history, physical examination, bone age X-ray, laboratory tests, and essential assessments mentioned above. Benign variants such as premature thelarche and premature adrenarche must be identified first.

**Table 5.** Physical examination of precocious puberty

Physical examination	Details	Reasons
Breast	According to Tanner staging	Assess breast development
Testes	Evaluate size using Prader orchidometer	Assess testicular development to differentiate causes
Penis	According to Tanner staging	Assess penile development
Clitoris	Measure length in centimeters	Assess clitoral development, especially in heterosexual precocity cases
Pubic hair	According to Tanner staging	Assess pubic hair development
Vulvar mucosa	Color of mucosa	Assess vulvar mucosa appearance which reflect estrogenic effect
Rash	Check for café-au-lait spots (irregular border)	Consider McCune Albright syndrome
Visual field	Assess vision	Pituitary gland tumors may affect vision
Abdominal mass	Palpate abdominal area	Detect abdominal tumors capable of hormone production
Dysmorphic features	All dysmorphic features should be observed and recorded	Pallister–Hall syndrome, William syndrome, Temple syndrome are the examples of syndrome associated with precocious puberty

In the first step of differential diagnosis, it is crucial to determine whether the early puberty is isosexual or heterosexual precocity. Isosexual precocity means that the development aligns with the individual’s sex. For example, girls show breast development, and boys exhibit testicular enlargement, indicating isosexual precocity. If the development is not consistent with the individual’s sex, such as clitoral enlargement in girls, it is termed heterosexual precocity, indicating peripheral precocity. If isosexual precocity is confirmed, further investigation is necessary to determine if it is central or peripheral precocity (Tables 6 and 7).

**Table 6.** Etiology of CPP [18]

System	Diseases
CNS lesions	Congenital
	– Hypothalamic hamartoma – Suprasellar arachnoid cysts – Hydrocephalus – Glioma or neurofibromatosis type 1 – Tuberous sclerosis – Septo-optic dysplasia – Chiari malformations and myelomeningocele Acquired – Tumors: astrocytoma, ependymoma, pinealoma, hypothalamic or optic glioma – Post-insults (perinatal, infection, trauma, radiotherapy) – Granulomatous disease – Cerebral palsy
No CNS lesions	– Idiopathic – Endocrine disruptors – Genetic changes: gain-of-function mutations in the genes encoding KISS1 and KISS1R: formerly called GPR54: G-protein coupled receptor, loss-of-function mutation in MKRN3 and DLK1 – Early exposure to sex steroids (secondary CPP)

1 CNS, central nervous system; CPP, central precocious puberty; DLK1, delta-like homolog 1; KISS1, kisspeptin; KISS1R, kisspeptin receptor, MKRN3, makorin ring finger 3.

**Table 7.** Etiology of PPP [19]

System	Diseases
Boys	–Beta-HCG secreting tumor (CNS/outside CNS) – Androgen secretion by adrenal glands and testes –CAH – Virilizing adrenal neoplasm – Leydig cell tumor – Familial testotoxicosis
Girls	– Ovarian cyst – Estrogen secreting ovarian or adrenal glands
Both sex	– MAS (PPP, irregular border café au lait spot & fibrous dysplasia) – Hypothyroidism – Exogenous sex steroid

1 CAH, congenital adrenal hyperplasia; CNS, central nervous system; HCG, human chorionic gonadotropin; MAS, McCune-Albright syndrome; PPP, peripheral precocious puberty.

**Figure 2.**
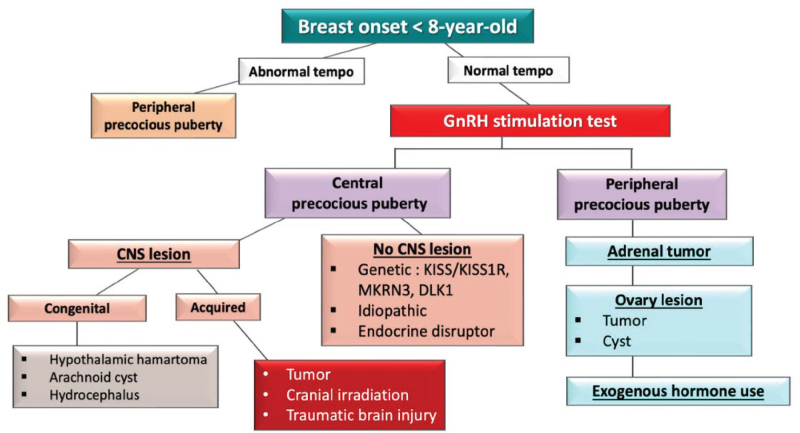
Diagnostic guidelines for precocious puberty in girls (MAS). CNS, central nervous system; DLK1, delta-like homolog 1; GnRH, gonadotropin-releasing hormone; KISS1, kisspeptin; KISS1R, kisspeptin receptor; MAS, McCune-Albright syndrome; MKRN3, makorin ring finger 3.

In the diagnosis in girls, evaluating the tempo of puberty is essential. If there are minimal breast developments but menarche has occurred, it may indicate peripheral precocity. However, if the sexual development follows a normal or increased tempo, a GnRH stimulation test is performed. Peripheral precocity may be due to ovarian cysts or exogenous hormone use. In cases of central precocity, additional neurological evaluation is needed to identify potential brain abnormalities. If no neurological issues are found, consider genetic or idiopathic causes (Figure 2, Table 8).

**Table 8.** Genetic mutation in CPP

Gene	Protein	Diseases
*MKRN3* [20]	MKRN3	Suppression of GnRH secretion
*KISS1*	KISS1	Disruption of puberty regulation
*KISS1R* [21]	KISS1 receptor	Impair response to KISS1 signaling
*DLK1* [5]	DLK1	Imprinting disorder affecting pubertal timing
*LIN28B*	Lin 28 Homolog B	Unknown, homolog of *Caenorhabditis elegans* protein may play in GnRH secretion
*NPYR1*	NPYR1	Inhibitory effect on GnRH pulse activity
*TAC3* [22]	Neurokinin B	Play role in GnRH release
*TACR3*	Neurokinin B receptor	Neurokinin receptors, which is G protein-coupled receptor that bind to neurokinin B may play a role in GnRH release.
*GABRA1* [23]	Gamma amino butyric acid A1 receptor α-1 subunit	GABA-A receptor α-1 subunit that binds to GABA may inhibit GnRH release.

1 CPP, central precocious puberty; DLK1, delta-like homolog 1; GABA, gamma-aminobutyric acid; GABRA1, gamma-aminobutyric acid receptor subunit alpha 1; GnRH, gonadotropin-releasing hormone; KISS1, kisspeptin; KISS1R, kisspeptin receptor; LIN28B, lin-28 homolog B; MKRN3, makorin ring finger protein 3; NPYR1, neuropeptide Y; TAC3, tachykinin 3; TACR3, tachykinin receptor 3.

**Figure 3.**
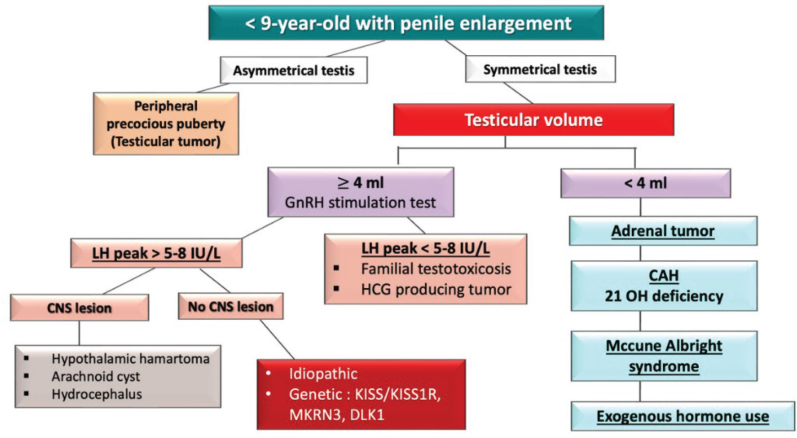
Diagnostic guidelines for precocious puberty in boys. CAH, congenital adrenal hyperplasia; CNS, central nervous system; DLK1, delta-like homolog 1; GnRH, gonadotropin-releasing hormone; KISS1, kisspeptin; KISS1R, kisspeptin receptor; LH, luteinizing hormone; MKRN3, makorin ring finger 3.

The diagnosis of diseases in young boys requires careful examination of the testes. If there are asymmetrical testes, PPP is suspected, caused by growths capable of hormone production near the testicles. In cases of symmetrical testes, attention is turned to the size of the testes. If the size is smaller than 4 mL, PPP is suspected, which could be due to an adrenal tumor, 21-hydroxylase deficiency congenital adrenal hyperplasia (CAH), or the use of exogenous hormones. If the testes are >4 mL, a GnRH stimulation test is conducted. In PPP, hormones might originate from beta-HCG producing tumor or from familial testotoxicosis, caused by activating mutations in LH receptors. If CPP is indicated, further investigation is necessary to determine if the patient has abnormalities in the nervous system. If no abnormalities are found, it could be due to genetic factors or idiopathic causes (Figure 3).

## Treatment of precocious puberty

### CPP

Gonadotropin-releasing hormone agonists (GnRHa) serve as the standard treatment for CPP. The mechanism involves downregulating GnRH receptors, suppressing the HPG axis. Treatment goals include preventing psychological issues related to precocious puberty and optimizing final adult height potential. Lowering sex hormones from gonads halts or slows pubertal maturation, delays epiphyseal plate closure, and enhances final height outcomes. Treatment may not be necessary for all CPP children if they achieve their target height. Monthly GnRHa injections have been approved for over 3 decades in CPP children, with most studies focusing on girls. Reports indicate height gains of 4.5–9.8 cm compared with predicted adult height (PAH) before treatment initiation [24, 25]. Although there are numerous varieties of GnRH agonists, the effectiveness of 1-monthly compared with 3-monthly leuprolide acetate is still restricted. Our previous study showed that the 3-monthly leuprolide acetate treatment showed greater hormonal and growth suppression effects, but there was no significant difference in PAH between the two groups [26, 27, 28]. A comprehensive review by Bereket et al. in 29 studies showed that CPP girls treated with GnRHa gained a final adult height 2–10.5 cm higher than predicted, averaging 4 cm. Factors predicting better final height include earlier chronological age, younger bone age at treatment start, greater height at diagnosis, and greater target height [26]. Height gain is greater with treatment initiation at <6–7 years of age [25]. Prolonged GnRHa use has no negative pregnancy outcomes compared with the controls, with similar unassisted pregnancy rates [29]. Transient weight gain during GnRHa therapy has been reported but normalized after discontinuation [30]. Long-term outcomes of a 12-weeks GnRHa depot formulation are rarely reported.

The combination of GH and GnRHa has been studied in precocious puberty girls with slow height velocity and poor predicted height. Tuvemo et al. [31] reported a higher adult height (158.9 cm) with combined GH/GnRH analog treatment compared with the GnRH analog-treated group (155.8 cm). By contrast, a Korean study showed no statistically significant difference between these groups, highlighting financial considerations due to the therapy’s expense.

Common side effects of GnRH agonist injections include pain at the injection site, hot flushes, and the potential development of sterile abscesses after injections. In long-term studies, the use of GnRH agonists in treatment has proven beneficial for increasing final height. It helps reduce the stress experienced by adolescents transitioning into puberty and does not affect the increase in body mass index, the decrease in bone density, or the development of polycystic ovarian syndrome.

### PPP

PPP occurs when excess sex hormones are produced by sources outside the hypothalamus–pituitary axis, such as tumors or adrenal glands. Treatment for PPP focuses on addressing the underlying cause and managing the effects of the excess hormones.

- *Surgical removal of tumors*: If a tumor is identified as the source of the excess hormones, surgical removal is crucial to eliminate the source of the problem. This is a common treatment for PPP caused by tumors in the ovaries, testes, or adrenal glands.
- *Corticosteroid hormone replacement*: In cases of PPP caused by CAH, corticosteroid hormone replacement therapy is used to suppress the production of androgens by the adrenal glands. This helps to regulate hormone levels and reduce the effects of precocious puberty.
- *Aromatase inhibitors*: Aromatase inhibitors are medications that block the enzyme aromatase, which is responsible for converting androgens into estrogens. These medications are particularly useful in girls with MAS, a condition that can cause early puberty and excessive bone growth. By inhibiting androgen conversion, aromatase inhibitors can help slow down the progression of puberty and reduce the risk of premature bone fusion.
- *Tamoxifen:* Tamoxifen is a medication that can act as an anti-estrogen or an anti-androgen, depending on the tissue type. It can be used as an adjuvant therapy in some cases of PPP, particularly when other treatments have not been fully effective.

## Conclusion

Precocious puberty requires detailed medical history, physical examinations, and laboratory tests. General practitioners and pediatricians need to consider this condition in children displaying signs of early puberty for accurate diagnosis and timely treatment. Appropriate and timely treatment can prevent complications and improve the quality of life for affected children.
